# Carboxyl Amidation of Chlorogenic Acid Improves Anti-Inflammatory Activity and Biosafety via Potent AKR1B1 Inhibition in LPS-Induced Macrophages

**DOI:** 10.3390/molecules31183247

**Published:** 2026-09-14

**Authors:** Faliu Yang, Chunmei Guang, Huan Chen, Hongwei Hou

**Affiliations:** Beijing Life Science Academy, Beijing 102200, China; yangfaliu@blsa.com.cn (F.Y.); guangcm@blsa.com.cn (C.G.)

**Keywords:** chlorogenic acid amide, carboxyl-amidation, AKR1B1 inhibition, anti-inflammation, RAW264.7 macrophages, molecular docking, cellular safety

## Abstract

Persistent inflammation driven by excessive AKR1B1 activation contributes to the progression of multiple chronic inflammatory diseases. Natural chlorogenic acid (CGA) exhibits anti-inflammatory activity but suffers from poor membrane permeability, while its laurate ester derivative (CGL) shows clear dose-dependent cytotoxicity in macrophages. Here, we synthesized a novel chlorogenic acid amide derivative (CGAA) by selective amidation of the aliphatic carboxyl group on the quinic acid moiety of CGA, fully preserving the chiral polyphenol scaffold. Structural identity was confirmed by HSQC, HMBC, FTIR and HR-ESI-MS. CCK-8 assays demonstrated that CGAA maintained > 95% viability of RAW264.7 cells at 100 μM, displaying markedly superior cellular safety relative to CGL. In LPS-stimulated RAW264.7 macrophages, CGAA dose-dependently suppressed NO, TNF-α and IL-6 production and exhibited stronger antioxidant activity than both CGA and CGL. Temperature-gradient CETSA, DARTS and enzymatic assays established direct binding and potent inhibition of AKR1B1 by CGAA (IC_50_ = 7.85 μM). Molecular docking revealed that the newly introduced amide forms an additional hydrogen bond with ILE-260, enhancing ligand–target affinity. This carboxyl-amidation strategy simultaneously improves the cellular safety and AKR1B1 inhibitory potency of CGA, providing a rational approach for developing low-toxicity polyphenol-based anti-inflammatory leads.

## 1. Introduction

Inflammation is an essential physiological defense against exogenous infection and tissue injury [1,2,3]. However, persistent low-grade inflammation contributes to the pathological progression of multiple chronic diseases, including diabetic complications, cardiovascular disorders and neurodegenerative lesions [4]. As key effector cells in inflammatory cascades, M1-polarized macrophages secrete large amounts of nitric oxide (NO), reactive oxygen species (ROS), TNF-α and IL-6 upon lipopolysaccharide (LPS) stimulation, thereby exacerbating tissue damage [5,6]. Targeting macrophage-mediated inflammatory pathways has therefore emerged as a promising therapeutic strategy for chronic inflammatory disorders [4].

Chlorogenic acid (CGA), a ubiquitous dietary polyphenol, exhibits well-documented anti-inflammatory and antioxidant bioactivities [7,8,9,10]. Its clinical translation is severely limited by an imbalanced lipophilic–hydrophilic profile. CGA shows moderate aqueous solubility but extremely weak lipophilicity, resulting in poor transmembrane permeability and low intracellular uptake [11]. In addition, its unsatisfactory chemical stability and rapid metabolic clearance further reduce bioavailability [9]. Structural modification of functional groups has therefore become a mature strategy to optimize the physicochemical properties and bioavailability of natural polyphenols [12].

Esterification is the predominant modification strategy reported for CGA, exemplified by chlorogenic acid laurate (CGL) [13]. Although alkyl ester substitution moderately increases lipophilicity and facilitates cell penetration, this modification introduces clear concentration-dependent cytotoxicity in macrophages. Moreover, CGL only achieves marginal improvements in anti-inflammatory activity and AKR1B1 inhibitory potency compared with unmodified CGA [13,14]. Thus, conventional ester derivatization fails to simultaneously optimize cellular biosafety and target-suppressive efficacy.

Amidation is a widely adopted scaffold optimization strategy in modern drug discovery. Amide linkages effectively balance lipophilicity, enhance chemical stability, reduce non-specific toxicity and strengthen ligand–protein non-covalent interactions [12,15]. Existing chlorogenic acid amide derivatives are predominantly generated by modification of phenolic hydroxyl groups on the caffeoyl moiety; such transformations disrupt the core polyphenolic scaffold responsible for anti-inflammatory activity [15]. A limited number of amides modified at the quinic acid carboxyl group have been reported, but these analogs were evaluated only for hypoglycemic activity against α-amylase and α-glucosidase [14]. To date, no systematic study has simultaneously assessed the AKR1B1 inhibitory capacity, macrophage anti-inflammatory effects and cellular biosafety of quinic acid-selective CGA amides, representing a clear research gap addressed by the present work.

Aldose reductase AKR1B1 acts as a pivotal upstream mediator of inflammatory signaling [16,17]. Excessive AKR1B1 activation triggers intracellular ROS accumulation and subsequent release of pro-inflammatory cytokines [18]. Epalrestat, a clinically approved AKR1B1 inhibitor, effectively alleviates LPS-induced macrophage inflammation, supporting the therapeutic value of AKR1B1 antagonists. Previous studies have confirmed only weak AKR1B1 suppression by native CGA, yet the direct binding mode and corresponding structure–activity relationship remain unclear [19].

A recent study investigated the AKR1B1 binding and anti-inflammatory effects of native CGA isomers in RAW264.7 macrophages, but did not involve structural modification or biosafety evaluation of derivatives [19]. Wang et al. previously synthesized quinic acid carboxyl CGA amides, yet only assessed their hypoglycemic activity against carbohydrate hydrolases, without systematic characterization of cellular anti-inflammation or AKR1B1 inhibition [14]. Distinct from these prior works, the present study selectively generates CGAA while retaining the intact polyphenolic scaffold, and simultaneously compares its target affinity, antioxidant capacity and macrophage biosafety against the toxic ester derivative CGL, thereby filling the unaddressed research gap.

**Figure 1:**
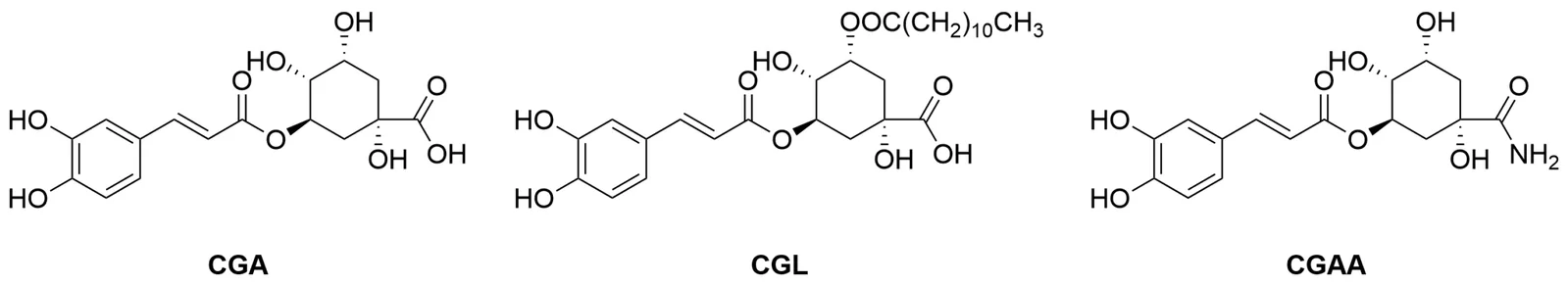
Chemical structures of chlorogenic acid (CGA), chlorogenic acid laurate (CGL), and the newly synthesized chlorogenic acid amide derivative (CGAA).

Herein, we selectively synthesized a chlorogenic acid amide derivative (CGAA) via one-step amidation at the free aliphatic carboxyl group on the quinic acid ring of CGA, without disrupting the polyphenol framework or inducing chiral racemization (Figure 1). We systematically compared the cellular biosafety, anti-inflammatory and antioxidant performance of CGAA with parent CGA and esterified CGL in LPS-stimulated RAW264.7 macrophages. Integrated spectroscopic characterization, intracellular target-binding assays (CETSA and DARTS), in vitro enzyme inhibition tests and molecular docking simulations were performed to clarify the direct interaction between CGAA and AKR1B1, and to elucidate the structure–activity benefits of carboxyl-amide modification. This study provides a rational skeleton-retaining derivatization strategy for natural CGA and offers a low-toxicity, high-potency AKR1B1 inhibitor candidate for the intervention of inflammatory diseases.

## 2. Results

### 2.1. Structural Characterization of CGAA

**Figure 2:**
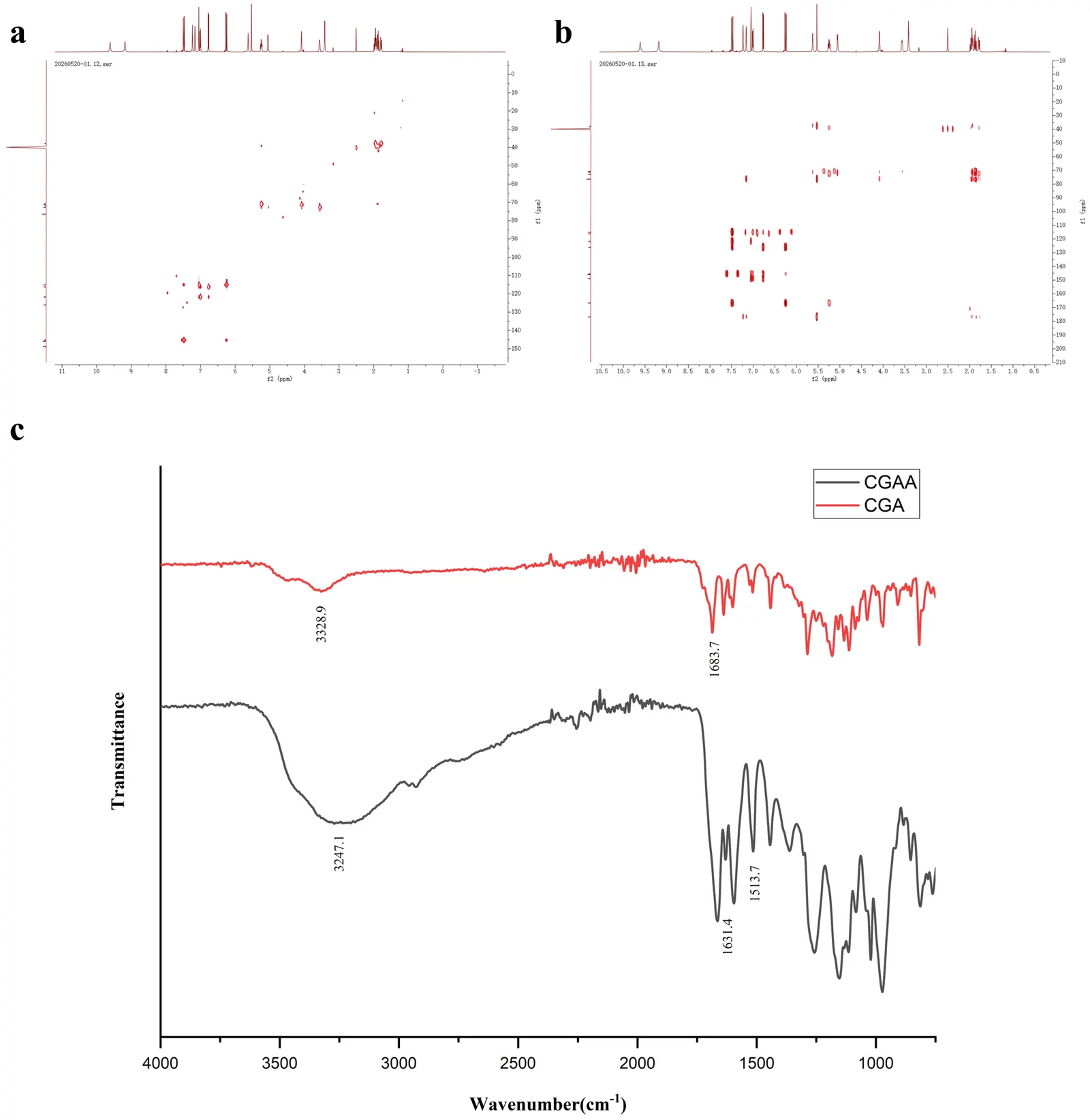
Structural characterization of synthetic CGAA. (**a**) HSQC two-dimensional NMR spectrum for full C-H assignment of CGAA skeleton. (**b**) HMBC spectrum showing diagnostic cross-peaks to locate the amidation modification site. (**c**) Comparative FTIR spectrum of native CGA and CGAA with key characteristic absorption bands labeled, which directly verify the successful formation of amide linkage.

CGAA was synthesized through selective amidation of the free aliphatic carboxyl group on the quinic acid moiety of chlorogenic acid (CGA). FTIR, HSQC, and HMBC analyses were performed to verify the formation of the amide linkage and to determine the modification site while assessing whether the native CGA framework remained intact (Figure 2).

The FTIR spectra of CGA and CGAA exhibited distinct differences consistent with successful amidation (Figure 2c). In CGAA, a broad absorption band at 3247.1 cm^−1^ was assigned to overlapping O–H and N–H stretching vibrations, and a weak band at 2929.2 cm^−1^ corresponded to aliphatic C–H stretching. The appearance of characteristic amide I (1631.4 cm^−1^) and amide II (1513.7 cm^−1^) bands, together with the disappearance of the carboxylic C=O stretching band of native CGA at 1683.7 cm^−1^, confirmed the conversion of the quinic acid carboxyl group into an amide functionality. The aromatic C=C vibration of the caffeoyl moiety remained at 1595.8 cm^−1^, and the C–O stretching bands in the 1000–1250 cm^−1^ region were preserved, indicating that the polyphenolic caffeoyl scaffold was not altered during the reaction.

Two-dimensional NMR spectroscopy further established the modification site. The HSQC spectrum (Figure 2a) showed complete C–H correlations corresponding to both the caffeoyl aromatic region and the quinic acid aliphatic carbons, consistent with the carbon framework of CGA. In the HMBC spectrum (Figure 2b), clear long-range correlations between the amide NH_2_ protons and the quinic acid carboxyl carbon demonstrated that amidation occurred specifically at the aliphatic carboxyl group, whereas the phenolic hydroxyl groups remained unmodified. Detailed spectroscopic data are provided in Section 4.2 and the Supplementary Materials.

Collectively, the FTIR and two-dimensional NMR data confirmed the successful synthesis of CGAA through selective amidation of the quinic acid carboxyl group while preserving the native caffeoyl polyphenol framework and stereochemical integrity of CGA. After confirming the chemical identity of CGAA, its cellular biosafety was compared with those of CGA and the esterified derivative CGL in RAW264.7 macrophages.

### 2.2. CGAA Exhibits Superior Cellular Biosafety Compared with CGA and CGL

**Figure 3:**
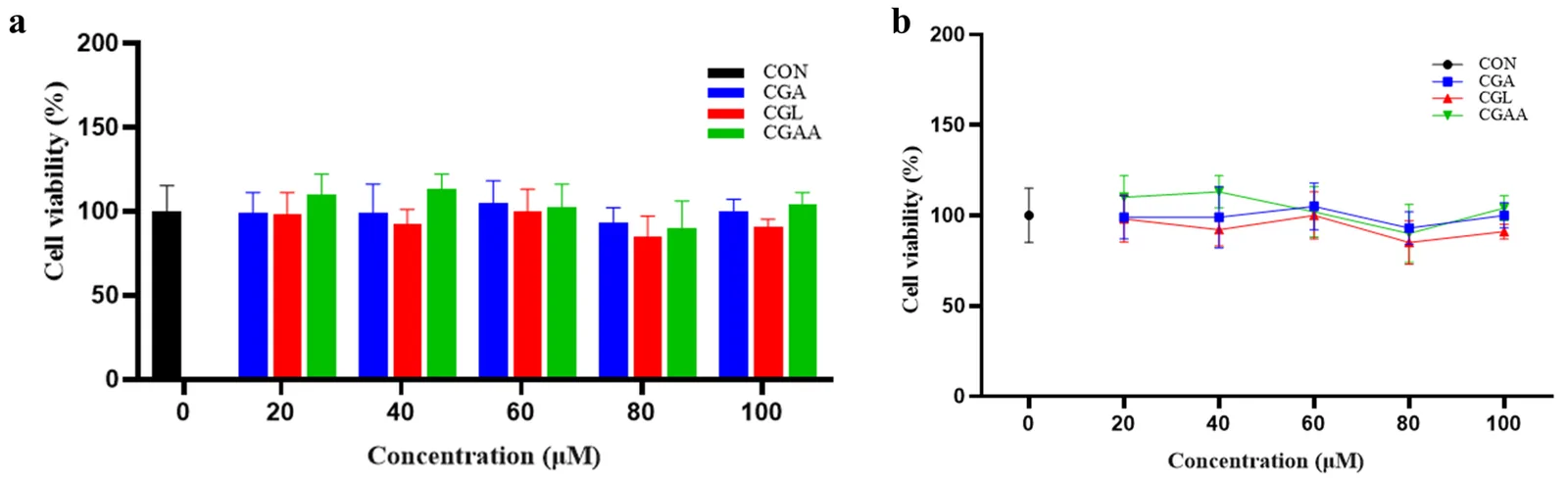
Cytotoxicity profiles of CGAA, CGA, and CGL in RAW264.7 macrophages after 24 h incubation. (**a**) Bar graph showing cell viability at serial concentrations from 0 to 100 μM. (**b**) Line plot showing the dose-dependent cytotoxic trend of the three derivatives. CGL induced a slight reduction in cell viability at 100 μM, whereas CGA and CGAA both exhibited favorable cellular compatibility. CGAA maintained cell viability above 95% at 100 μM and exhibited the optimal biosafety profile. All data are expressed as mean ± SD (*n* = 3). Statistical analysis was performed by one-way ANOVA with Tukey’s post hoc test. No statistically significant differences in cell viability were observed among the three compounds at any of the tested concentrations (*p* > 0.05).

The cytotoxicity of CGAA, native CGA, and the esterified derivative CGL was initially screened over a broad concentration range (10–1280 μM) in RAW264.7 macrophages using the CCK-8 assay. Because CGL exhibited more noticeable cytotoxicity at concentrations above 160 μM, a refined concentration series of 20, 40, 60, 80, and 100 μM was subsequently selected to define the non-cytotoxic concentration window for subsequent cellular experiments (Figure 3). Cell viability was assessed after 24 h of incubation to compare the cellular compatibility of the three compounds.

At the tested concentrations (≤100 μM), all three compounds maintained relatively high cell viability. CGL showed a slight reduction in cell viability at 100 μM, while native CGA and CGAA both retained viability above 90%. CGAA displayed the most favorable safety profile, with cell viability remaining above 95% even at 100 μM. Statistical analysis indicated no significant differences among the three compounds across the concentration range examined. Overall, the biosafety ranking under the non-cytotoxic window was CGAA > CGA > CGL.

These results indicate that carboxyl-to-amide modification maintains good cellular compatibility of the CGA scaffold while avoiding the more pronounced cytotoxicity associated with the long-chain ester derivative CGL at higher concentrations. The concentration of 100 μM was therefore established as the upper concentration limit for subsequent cellular assays.

### 2.3. CGAA Potently Suppresses LPS-Induced Oxidative Stress and Inflammatory Response

The antioxidant and anti-inflammatory activities of CGAA were systematically compared with those of native CGA and the esterified derivative CGL in LPS-stimulated RAW264.7 macrophages. LPS stimulation induced pronounced intracellular oxidative stress and inflammatory activation, whereas pretreatment with the three compounds attenuated these responses in a concentration-dependent manner, with CGAA exhibiting the strongest overall activity.

**Figure 4:**
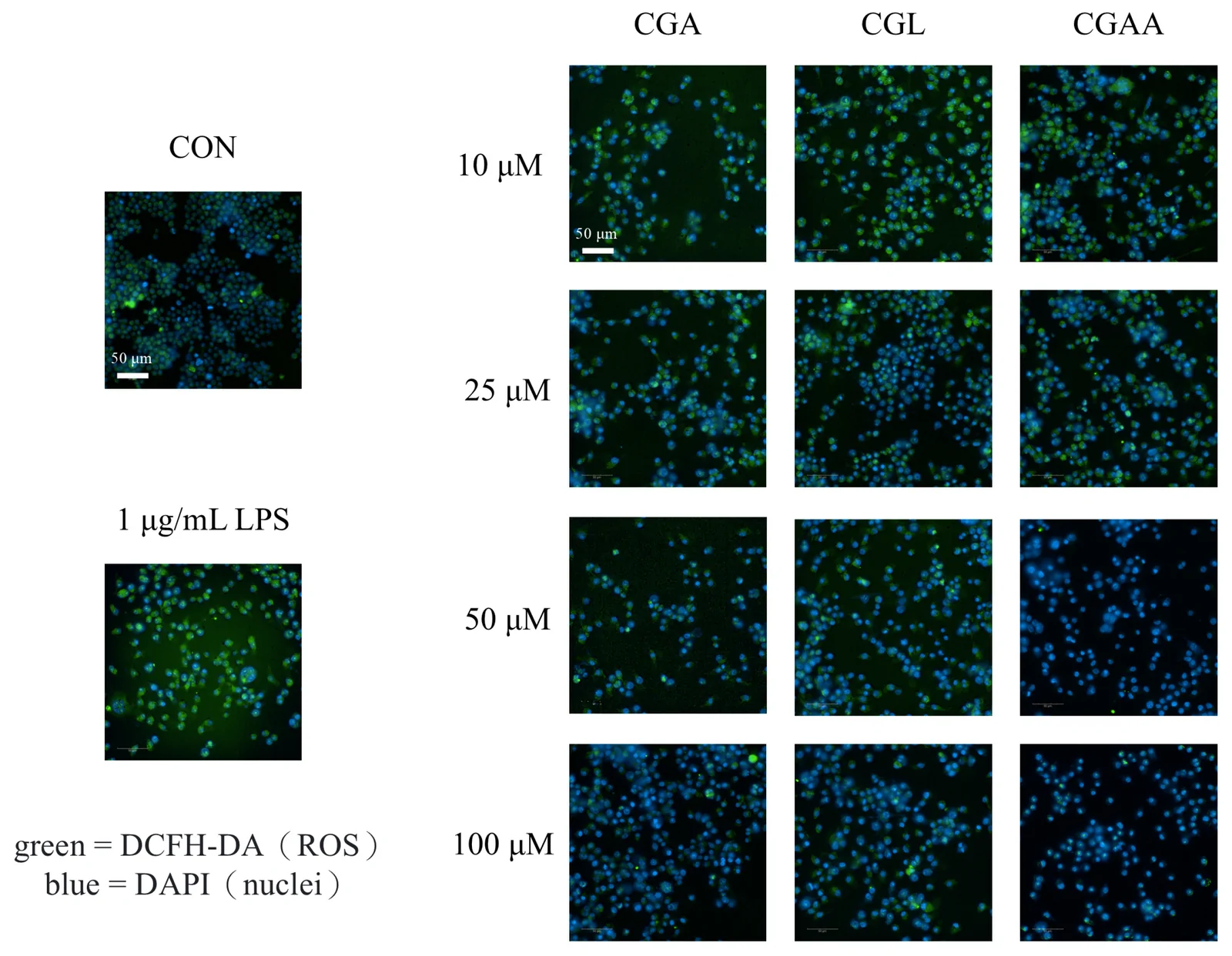
Representative fluorescence images of intracellular ROS production in RAW264.7 macrophages. Cells were pretreated with CGAA, CGA, or CGL at concentrations from 10 to 100 μM for 2 h, followed by stimulation with LPS (1 μg/mL) for 24 h. Intracellular ROS was stained with DCFH-DA (green fluorescence), and cell nuclei were counterstained with DAPI (blue fluorescence). Scale bar = 50 μm and applies to all panels. Quantitative analysis of fluorescence intensity is presented in Figure 5.

Intracellular ROS generation was first evaluated by DCFH-DA fluorescence staining (Figure 4). The LPS group displayed intense green fluorescence, indicating substantial ROS accumulation. Pretreatment with CGAA markedly reduced intracellular ROS levels in a concentration-dependent manner, and fluorescence intensity was strongly diminished at 100 μM. CGA also reduced ROS generation but was less effective than CGAA at equivalent concentrations, whereas CGL produced only a modest reduction in fluorescence across the tested concentration range.

**Figure 5:**
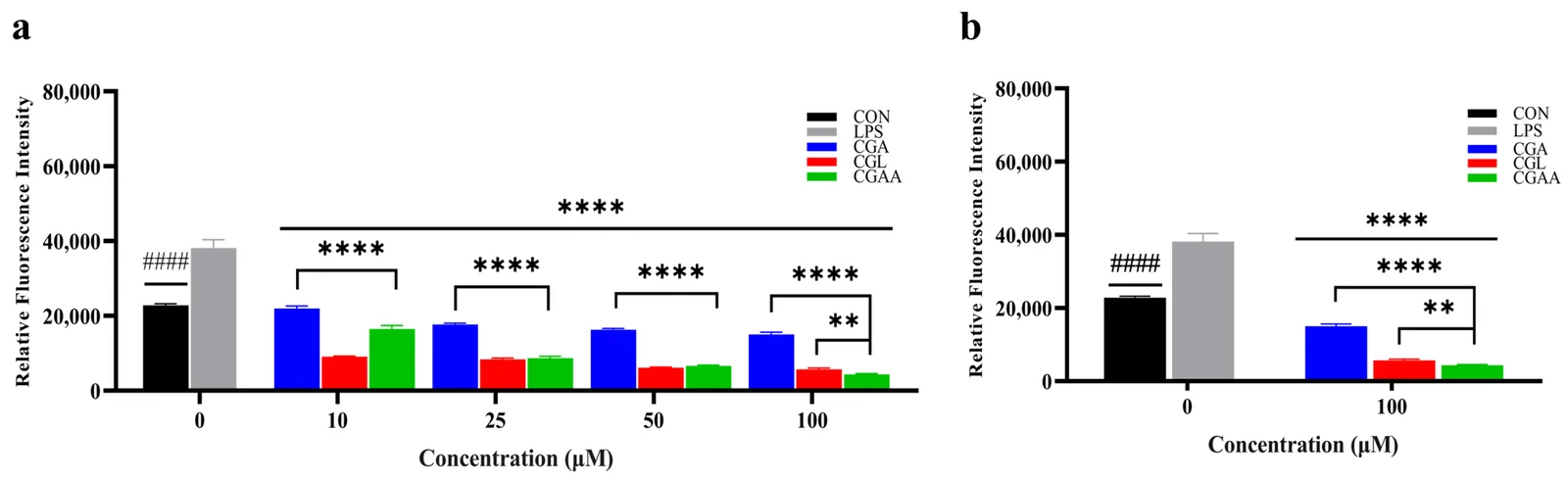
Quantitative analysis of LPS-induced intracellular ROS accumulation after CGAA, CGA, or CGL treatment in RAW264.7 macrophages. (**a**) Relative ROS fluorescence intensity at serial concentrations ranging from 10 to 100 μM. (**b**) Single-dose comparison of ROS levels at 100 μM. All data are presented as mean ± SD (*n* = 3). #### *p* < 0.0001 vs. control group; **** *p* < 0.0001, ** *p* < 0.01 vs. LPS group.

Quantitative analysis of intracellular ROS levels further confirmed the fluorescence imaging results (Figure 5). LPS treatment significantly increased ROS production compared with the untreated control group. All three compounds reduced ROS levels in a concentration-dependent manner, and the antioxidant activity followed the order CGAA > CGA > CGL at all corresponding concentrations. The single-dose comparison at 100 μM (Figure 5b) further demonstrated the superior ROS-scavenging capacity of CGAA.

**Figure 6:**
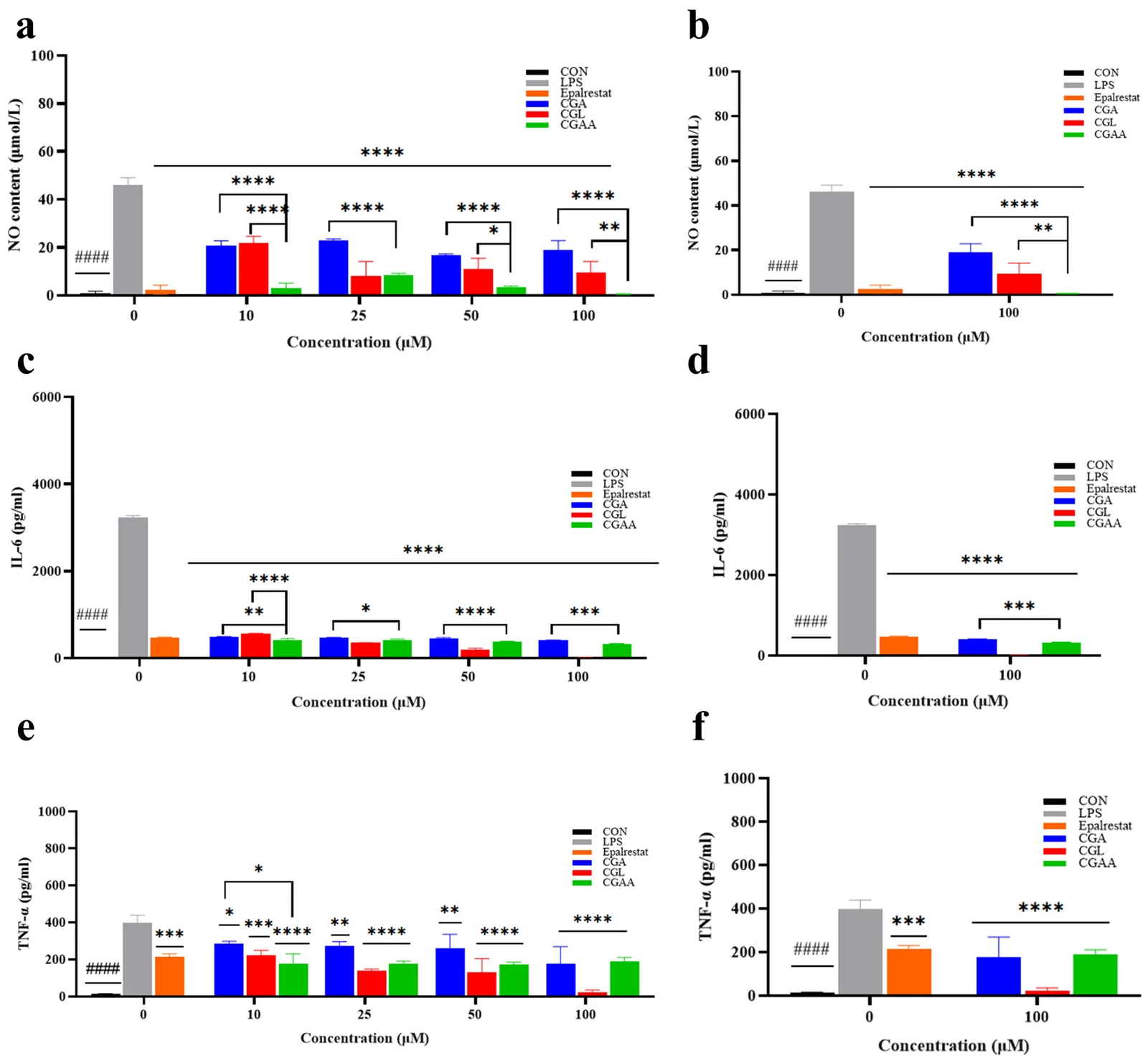
Quantitative detection of LPS-induced inflammatory mediators after treatment with CGAA, CGA, CGL and positive control epalrestat in RAW264.7 macrophages. (**a**,**b**) Determination of NO production: (**a**) Full concentration–response profiles at 10–100 μM. (**b**) Single-dose comparison at 100 μM. (**c**,**d**) Quantification of IL-6 secretion: (**c**) Full concentration–response profiles at 10–100 μM. (**d**) Single-dose comparison at 100 μM. (**e**,**f**) Quantification of TNF-α secretion: (**e**) Full concentration–response profiles at 10–100 μM. (**f**) Single-dose comparison at 100 μM. Cells were pretreated with the indicated compounds (10–100 μM) for 2 h, followed by LPS (1 μg/mL) stimulation for 24 h. NO levels were determined via the Griess assay, while IL-6 and TNF-α concentrations were quantified by ELISA. All data are expressed as mean ± SD (*n* = 3). #### *p* < 0.0001 vs. the control group; **** *p* < 0.0001, *** *p* < 0.001, ** *p* < 0.01, * *p* < 0.05 vs. the LPS group.

Nitric oxide (NO) production was subsequently measured as an indicator of macrophage inflammatory activation (Figure 6a,b). LPS stimulation markedly increased extracellular NO release, whereas pretreatment with CGAA, CGA, and CGL reduced NO production in a concentration-dependent manner. Among the three compounds, CGAA produced the greatest reduction in NO levels and restored NO production close to the basal level at 100 μM. The secretion of the pro-inflammatory cytokines IL-6 and TNF-α was further quantified by ELISA (Figure 6c–f). LPS significantly elevated the release of both cytokines, while all three compounds suppressed cytokine production in a concentration-dependent manner. CGAA consistently exhibited the strongest inhibitory effect across the tested concentration range, and at 100 μM its suppression of IL-6 and TNF-α was comparable to that of the positive control epalrestat and significantly greater than that of CGA and CGL.

Overall, these results demonstrate that CGAA effectively attenuates LPS-induced oxidative stress and inflammatory responses in RAW264.7 macrophages. Compared with native CGA and the esterified derivative CGL, CGAA produced the most pronounced reductions in intracellular ROS accumulation and in the release of NO, IL-6, and TNF-α, indicating superior antioxidant and anti-inflammatory activity under the experimental conditions tested.

### 2.4. CGAA Directly Binds to AKR1B1 Validated by CETSA and DARTS Assays

To clarify the molecular mechanism underlying the superior antioxidant and anti-inflammatory activity of CGAA observed in Section 2.3, we performed Cellular Thermal Shift Assay (CETSA) and Drug Affinity Responsive Target Stability (DARTS) assays to verify the direct physical interaction between the three compounds and AKR1B1 [20,21].

**Figure 7:**
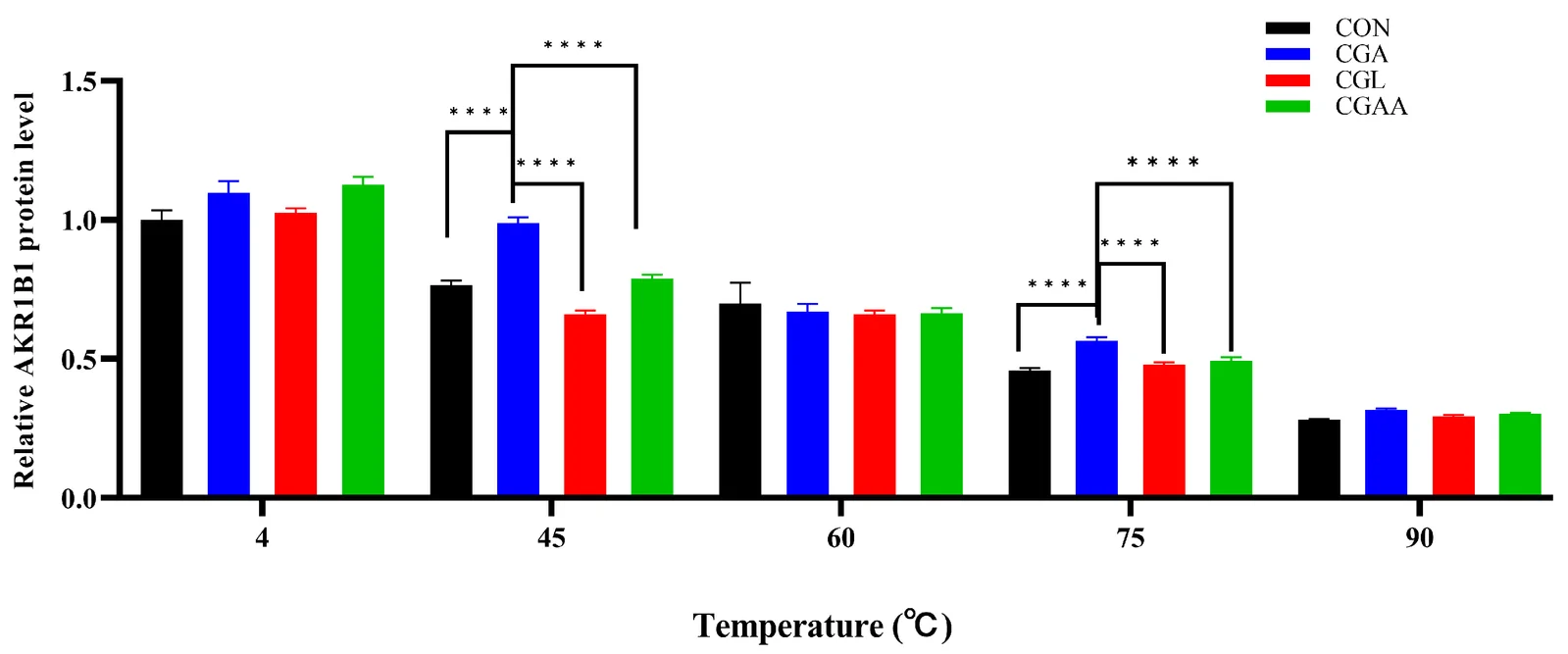
Temperature-gradient CETSA analysis of AKR1B1. Cell lysates pretreated with CGA, CGL, or CGAA were heated at 4, 45, 60, 75, or 90 °C. Residual soluble AKR1B1 protein was quantified by ELISA and normalized to the corresponding 4 °C sample. Data are presented as mean ± SD (*n* = 3). **** *p* < 0.0001 vs. the CGA group.

To further examine the direct interaction between the compounds and AKR1B1, a temperature-gradient cellular thermal shift assay (CETSA) was performed (Figure 7). Cell lysates were heated across a temperature range of 4–90 °C. Soluble AKR1B1 levels decreased progressively with increasing temperature in all groups, consistent with thermal denaturation. At intermediate temperatures, modest differences among the compounds were observed: CGL showed relatively lower residual protein levels at 60 °C, while CGAA and CGA exhibited thermal stability profiles closer to the vehicle control. Overall, none of the three compounds produced a dramatic, temperature-wide stabilization of AKR1B1. These results indicate that ligand-induced changes in thermal stability are moderate, and that differences in binding pose (rather than bulk thermal protection) may better explain the superior inhibitory potency of CGAA observed in the subsequent DARTS and enzymatic assays.

**Figure 8:**
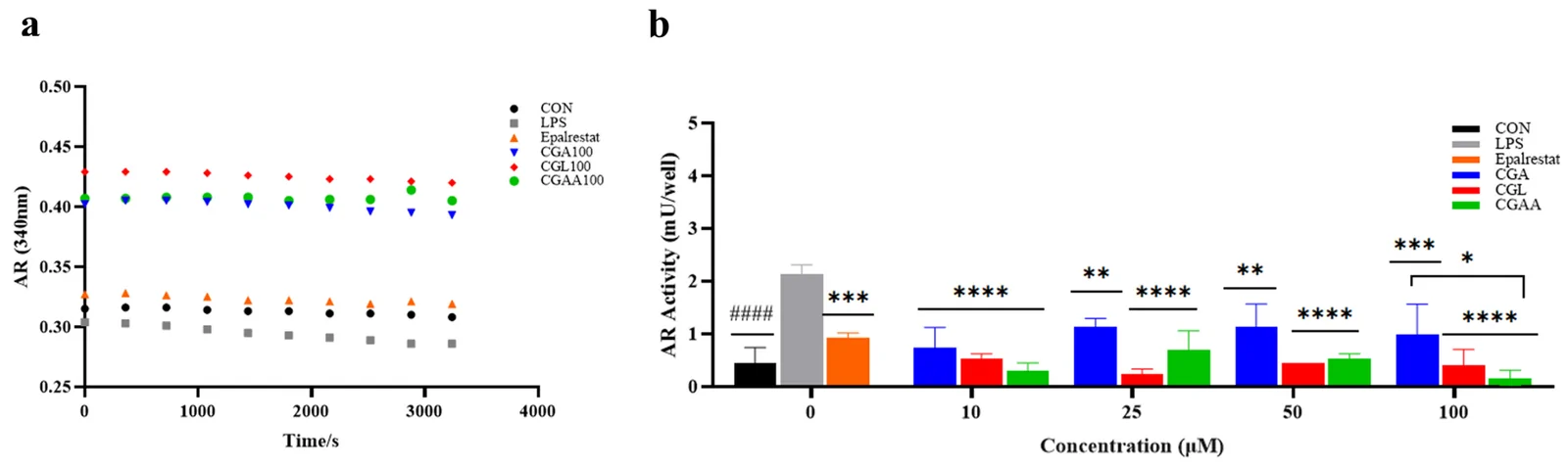
DARTS proteolysis assay verifying the direct binding of CGA, CGL and CGAA to AKR1B1. (**a**) Time-course residual AKR1B1 activity (monitored at 340 nm) after trypsin digestion in the presence or absence of compounds. (**b**) Dose-dependent protective effect of compounds against trypsin-induced AKR1B1 degradation at concentrations ranging from 10 to 100 μM. Recombinant AKR1B1 protein was pre-incubated with the indicated compounds prior to trypsin digestion, and residual AKR1B1 activity was detected via absorbance at 340 nm. All data are expressed as mean ± SD (*n* = 3). #### *p* < 0.0001 vs. the control group; **** *p* < 0.0001, *** *p* < 0.001, and ** *p* < 0.01 vs. the LPS group; * *p* < 0.05 vs. the CGAA group.

Consistent with the intracellular binding evidence from CETSA, cell-free DARTS assays further validated the direct interaction of the three compounds with AKR1B1 (Figure 8). CGAA exerted the strongest protective effect against trypsin digestion in a concentration-dependent manner, followed by CGA, while CGL showed the weakest protection. The binding potency ranking obtained from DARTS was therefore CGAA > CGA > CGL, which differed from the thermal stabilization ranking in CETSA. This divergence is expected, as the two assays report distinct aspects of protein–ligand interaction: CETSA primarily reflects thermal conformational stability, whereas DARTS evaluates resistance to protease cleavage [20].

The distinct binding behaviors of the three compounds arise from the different terminal functional groups on the quinic acid ring. The long alkyl ester chain of CGL provides limited polar interaction sites for non-covalent contacts with residues in the AKR1B1 binding pocket. Native CGA, bearing a free carboxyl group, forms moderate polar interactions. In contrast, the amide moiety of CGAA contains both hydrogen-bond donors and acceptors, enabling richer intermolecular interactions with key residues in the catalytic cavity [14,22]. Detailed differences in binding conformation will be further elucidated by molecular docking in Section 2.6.

Collectively, intracellular CETSA and cell-free DARTS assays jointly confirmed that CGAA acts as a direct binding ligand of AKR1B1. Notably, the ranking of thermal stabilization (CGA > CGAA > CGL) differed from the ranking of protease resistance and enzymatic inhibition (CGAA > CGA > CGL). This discrepancy is expected, because CETSA primarily reflects ligand-induced thermal conformational stability, whereas DARTS and enzyme activity assays report the functional consequences of the binding pose within the catalytic pocket. The superior catalytic inhibition by CGAA indicates that its amide-mediated interactions more effectively obstruct substrate/NADPH binding, even though the overall thermal stabilization effect is moderate. These functional advantages fully account for the potent anti-inflammatory and antioxidant effects observed in the cellular assays described in Section 2.3.

### 2.5. CGAA Inhibits AKR1B1 Enzyme Activity with Higher Potency than CGA and CGL

In vitro recombinant AKR1B1 enzymatic assays were performed to quantitatively compare the catalytic inhibitory capacity of CGAA, native CGA and esterified CGL, and to calculate the corresponding IC_50_ values as a direct functional indicator of target suppression.

All three compounds suppressed AKR1B1 catalytic activity in a clear concentration-dependent manner. Non-linear regression fitting with GraphPad Prism 9 yielded IC_50_ values of 7.85 μM for CGAA, 15.62 μM for CGA and 28.37 μM for CGL. The inhibitory potency order was therefore CGAA > CGA > CGL, which was consistent with the protective ranking observed in DARTS assays but differed from the thermal stabilization ranking obtained from CETSA. This discrepancy further confirms that ligand-induced protein thermal stability cannot be simply equated to catalytic inhibition efficiency; the binding pose within the AKR1B1 catalytic pocket plays a decisive role in determining inhibitory activity.

**Figure 9:**
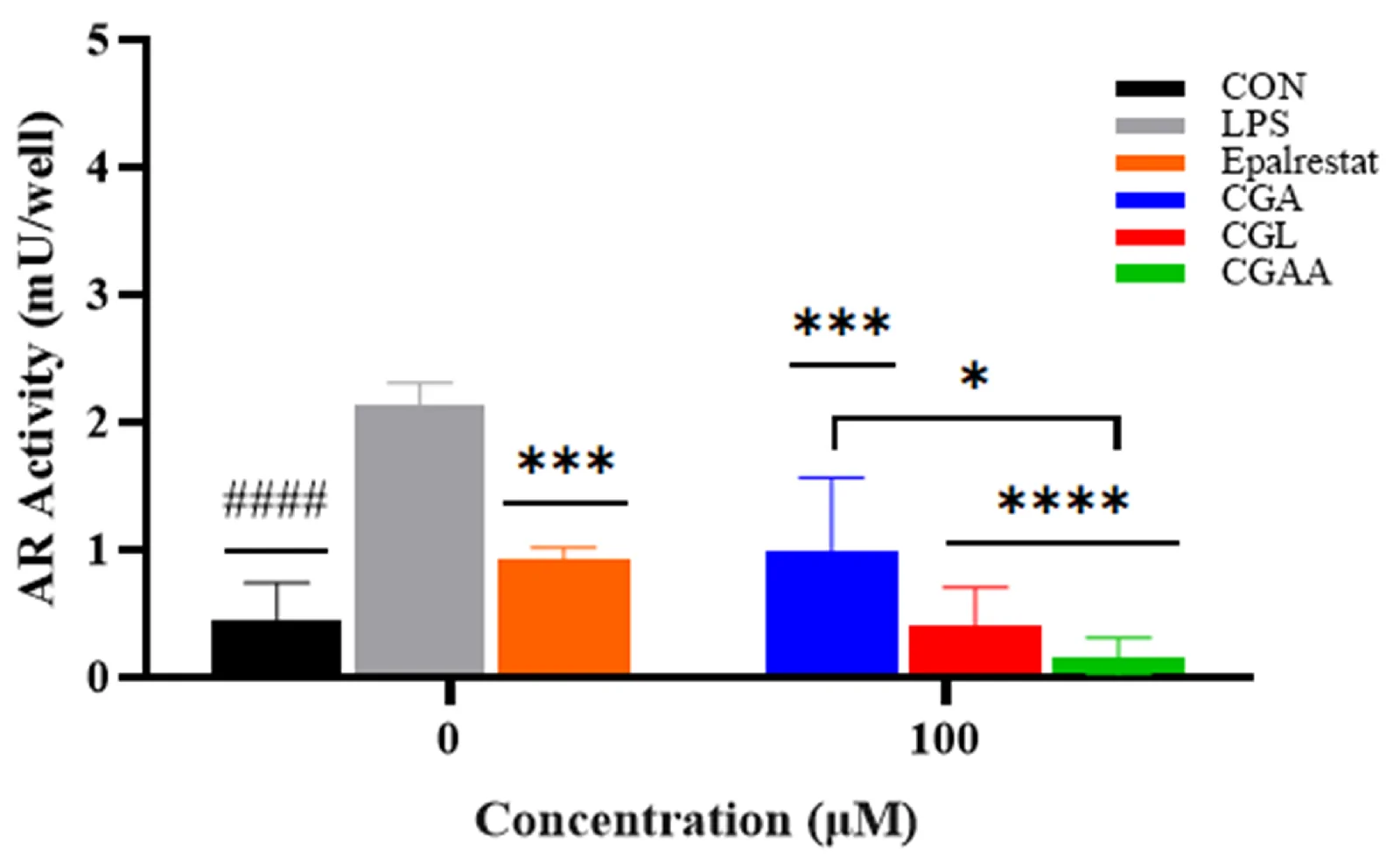
Comparative inhibitory effects of CGA, CGL and CGAA on AKR1B1 enzymatic activity at a fixed concentration of 100 μM. AKR1B1 activity was quantified by measuring absorbance at 340 nm. All data are expressed as mean ± SD (*n* = 3). #### *p* < 0.0001 vs. the control group; **** *p* < 0.0001 and *** *p* < 0.001 vs. the LPS group; * *p* < 0.05 vs. the CGAA group.

Single-dose comparison at 100 μM (Figure 9) further confirmed the superior AKR1B1 inhibitory effect of CGAA. At the same treatment concentration, CGAA reduced AKR1B1 activity to the lowest level, markedly outperforming both CGA and CGL. Combined with the CETSA and DARTS results in Section 2.4, these data indicate that replacement of the free aliphatic carboxyl group of CGA with an amide linkage strengthens favorable intermolecular interactions within the AKR1B1 catalytic cavity, thereby significantly enhancing catalytic inhibition. In contrast, the long alkyl ester chain of CGL disrupts the optimal binding conformation and weakens target inhibition. Taken together, carboxyl-amidation endows CGAA with the highest AKR1B1 antagonistic potency among the three compounds, which is consistent with its superior anti-inflammatory and antioxidant activity observed in RAW264.7 macrophages.

### 2.6. CGAA Exhibits Superior Binding Affinity and Unique Pocket Interaction with AKR1B1

**Figure 10:**
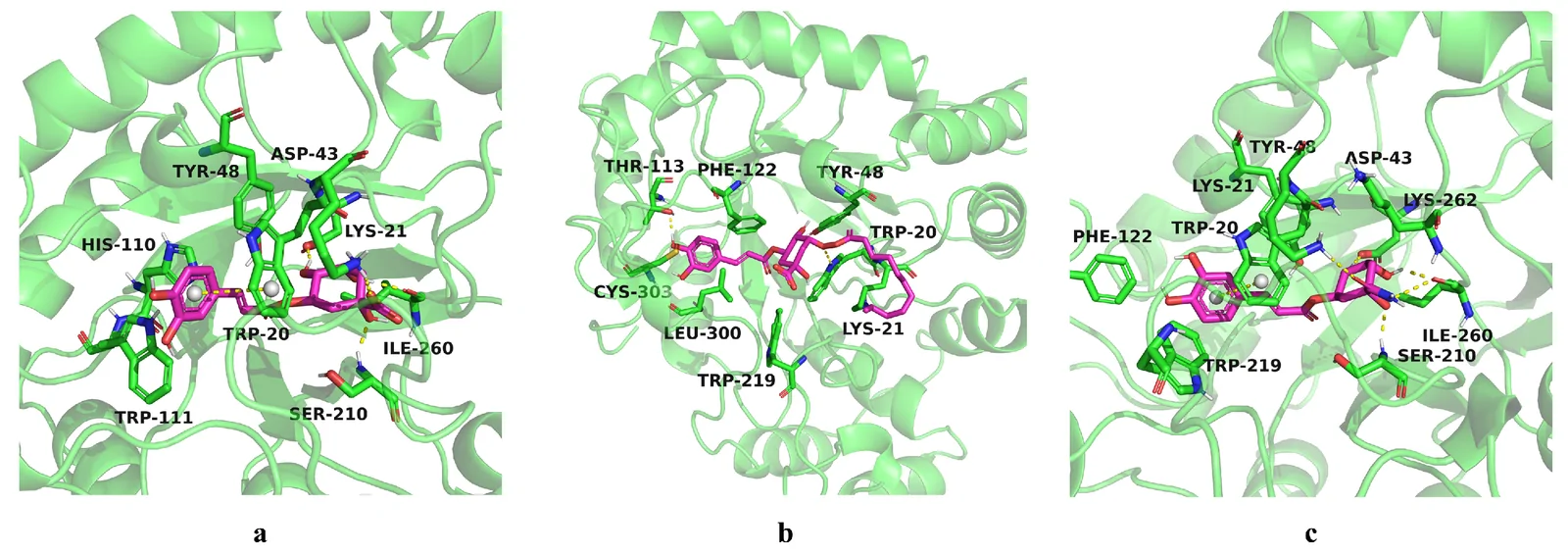
Molecular docking simulations of CGA, CGL and CGAA bound to human AKR1B1 protein. (**a**) Binding conformation of CGA: hydrogen bonds (yellow dashed lines) are formed with ASP-43, LYS-21, ILE-260 and SER-210, accompanied by a π–π stacking interaction with TRP-20 (gray spheres). (**b**) Binding conformation of CGL: its long alkyl ester side chain shifts the ligand pose, weakening polar hydrogen-bond interactions and strengthening hydrophobic contacts. (**c**) Binding conformation of CGAA: CGAA maintains all core intermolecular interactions of CGA, and its terminal amide group creates an additional hydrogen bond with ILE-260 to stabilize the ligand–protein complex. Left panels show the overall binding location of each ligand within the AKR1B1 pocket; magnified close-up views of key non-covalent interactions are displayed in the corresponding subgraphs.

Molecular Glide docking simulations were performed to elucidate the atomic-level structural basis for the divergent AKR1B1 inhibitory potencies of CGAA, CGA and CGL. The Glide binding free energy scores followed the order CGAA (−9.20) > CGA (−8.87) > CGL (−8.19), which was fully consistent with the IC_50_ ranking from the enzymatic assays. Detailed analysis of non-covalent interactions revealed clear structural differences among the three ligands (Figure 10).

CGA (Figure 10a) forms multiple hydrogen bonds (yellow dashed lines) with residues ASP-43, LYS-21, ILE-260 and SER-210, together with a π–π stacking interaction with TRP-20 within the anion-binding pocket. However, the free carboxyl group of CGA does not generate additional polar contacts that would lock the ligand into an optimal inhibitory conformation. CGL (Figure 10b) adopts an altered binding pose due to its long alkyl ester side chain. This positional shift disrupts part of the polar hydrogen-bond network inherited from parent CGA, and hydrophobic contacts become the dominant intermolecular force, accounting for its weaker target affinity and lower enzyme inhibitory activity. CGAA (Figure 10c) fully retains the core hydrogen-bonding pattern and π–π stacking interaction of CGA. Most importantly, the newly introduced amide group forms an additional hydrogen bond with ILE-260, which substantially stabilizes the ligand–AKR1B1 complex.

The AKR1B1 binding pocket consists of two characteristic subdomains: a conserved anion-binding region formed by TRP-20, TYR-48, HIS-110 and TRP-111, and a spacious hydrophobic specificity pocket lined by TRP-79, PHE-115, PHE-122, TRP-219, LEU-300 and CYS-303. Compared with CGL, CGAA formed stronger hydrophobic interactions with PHE-122 and TRP-219 within the hydrophobic pocket. Collectively, the additional hydrogen bond contributed by the amide moiety, together with the reinforced hydrophobic contacts, elevated the binding affinity of CGAA toward AKR1B1. This structural feature provides a direct explanation for the superior protease resistance and enzymatic inhibitory potency of CGAA observed in the preceding biochemical assays [22,23].

### 2.7. Parallel Anti-Inflammatory Comparison Between CGAA and Clinical AKR1B1 Inhibitor Epalrestat

To further evaluate whether AKR1B1 inhibition contributes to the anti-inflammatory activity of CGAA, epalrestat, a clinically approved AKR1B1 inhibitor, was included as a positive control for parallel comparison in the LPS-induced RAW264.7 macrophage model.

Epalrestat significantly reduced the LPS-induced secretion of TNF-α and IL-6 in RAW264.7 macrophages. Notably, CGAA exhibited anti-inflammatory activity comparable to that of epalrestat at the tested concentration, whereas native CGA and the esterified derivative CGL showed substantially weaker inhibitory effects on cytokine production. These results are consistent with the trends observed in the ROS, NO, and cytokine assays presented in Section 2.3.

Together with the CETSA, DARTS, enzymatic inhibition, and molecular docking results presented in Section 2.4, Section 2.5 and Section 2.6, the comparable anti-inflammatory efficacy of CGAA and epalrestat supports the involvement of AKR1B1 inhibition in the biological activity of CGAA. The selective carboxyl-amide modification of CGA therefore provides a derivative with enhanced anti-inflammatory efficacy relative to both the parent compound and the esterified analog.

## 3. Discussion

### 3.1. Structure–Activity Relationship and AKR1B1 Binding Mechanism

Previous structural modification of chlorogenic acid (CGA) has focused predominantly on esterification or derivatization of phenolic hydroxyl groups, which often compromises the intrinsic antioxidant activity of the polyphenol scaffold. In contrast, the present study employed selective amidation of the quinic acid carboxyl group while preserving the intact caffeoyl framework and native stereochemical configuration of CGA. This strategy differs from previously reported quinic acid-modified CGA amides that were mainly evaluated for carbohydrate-hydrolyzing enzyme inhibition, and provides direct evidence that selective carboxyl amidation can simultaneously improve cellular biosafety, anti-inflammatory activity, and AKR1B1 inhibitory potency in macrophages.

The comparative analysis of CGA, CGL, and CGAA revealed a clear structure–activity relationship. Native CGA retained favorable biocompatibility but exhibited only moderate antioxidant and anti-inflammatory activity, whereas esterification with a laurate chain improved lipophilicity at the cost of pronounced concentration-dependent cytotoxicity. In contrast, replacement of the free carboxyl group with an amide functionality generated a derivative that maintained excellent cellular compatibility while exhibiting markedly enhanced suppression of ROS accumulation, NO production, and pro-inflammatory cytokine secretion. These observations suggest that selective carboxyl amidation represents a more balanced optimization strategy than esterification for improving the pharmacological profile of CGA derivatives.

The mechanistic evidence obtained in this study supports AKR1B1 as a major molecular target of CGAA. Temperature-gradient CETSA showed that ligand-induced thermal stabilization of AKR1B1 was moderate for all three compounds, with only modest differences observed at intermediate temperatures. In contrast, CGAA exhibited clearly stronger protection against proteolytic degradation in DARTS and the lowest IC_50_ in the enzymatic assay. These findings reinforce that thermal stability ranking does not necessarily correlate with catalytic inhibition, and that the binding conformation within the active site is likely a more decisive factor for inhibitory potency.

Molecular docking analysis further explained the enhanced activity of CGAA at the structural level. The amide-modified derivative retained the core hydrogen-bonding network and π–π stacking interactions of native CGA while introducing an additional hydrogen bond with ILE-260 and strengthening hydrophobic interactions with residues in the specificity pocket. This optimized interaction network is consistent with the lower IC50 value of CGAA and its superior anti-inflammatory efficacy in LPS-stimulated macrophages. The higher cellular concentrations required for biological activity compared with the purified enzyme assay are likely attributable to membrane permeability barriers, intracellular protein binding, and metabolic consumption in the cellular environment.

Taken together, these findings demonstrate that selective amidation of the quinic acid carboxyl group constitutes an effective structure-guided optimization strategy for CGA. By preserving the native polyphenol scaffold while enhancing target interaction with AKR1B1, carboxyl amidation provides a rational approach for developing low-toxicity, high-potency polyphenol-based AKR1B1 inhibitors for inflammatory disease intervention.

### 3.2. Limitations and Future Perspectives

Despite the encouraging anti-inflammatory activity of CGAA, several limitations should be acknowledged. First, all biological evaluations were performed exclusively in the RAW264.7 murine macrophage cell line. The therapeutic efficacy, pharmacokinetic behavior, tissue distribution, metabolic stability, and potential off-target effects of CGAA remain to be validated in primary macrophages and, more importantly, in relevant in vivo models of inflammatory diseases (e.g., LPS-induced systemic inflammation, DSS-induced colitis, or other chronic inflammatory models) [2,24,25]. Second, although temperature-gradient CETSA, DARTS, enzymatic assays and molecular docking collectively support AKR1B1 as a direct molecular target, the observed thermal stabilization effects were only moderate. Further validation using additional orthogonal biophysical methods and cellular target-engagement assays would strengthen the mechanistic conclusions. Third, the present work does not exclude the possibility that additional inflammatory signaling pathways (such as NF-κB and MAPK) contribute to the observed biological effects; investigation of these pathways together with AKR1B1 knockdown or knockout models would provide stronger mechanistic validation.

In addition, the current study focused primarily on classical pro-inflammatory indicators (ROS, NO, TNF-α, and IL-6), whereas anti-inflammatory cytokines and macrophage polarization markers (M1/M2) were not examined. Comprehensive transcriptomic, proteomic, or metabolomic analyses could further clarify the broader regulatory network influenced by CGAA. Nevertheless, the present work demonstrates that selective carboxyl amidation of chlorogenic acid simultaneously improves cellular biosafety and AKR1B1 inhibitory potency while preserving the native polyphenol scaffold. These findings provide a rational structure-guided strategy for optimizing chlorogenic acid derivatives and support the further development of CGAA as a promising low-toxicity AKR1B1-targeted anti-inflammatory lead compound [26].

## 4. Materials and Methods

### 4.1. Materials and Apparatus

Chlorogenic acid (CGA, purity > 98%) was purchased from Bidepharm Co., Ltd. (Shanghai, China). Lauroyl chloride and triethylamine (TEA) were obtained from Sigma-Aldrich Corporation (St. Louis, MO, USA). 1-ethyl-3-(3-dimethylaminopropyl)carbodiimide hydrochloride (EDCI·HCl) and N-Hydroxybenzotriazole (HOBT) were supplied by Macklin Biochemical Co., Ltd. (Shanghai, China). Aqueous ammonia and other general analytical-grade chemical reagents were commercially available. Anhydrous N,N-dimethylformamide (DMF), tetrahydrofuran (THF), ethyl acetate, petroleum ether, acetone, n-hexane, ethanol and other analytical-grade solvents were used for synthetic procedures. Silica gel (200–300 mesh) for column chromatography was supplied by Aladdin Biochemical Technology Co., Ltd. (Shanghai, China).

RAW264.7 murine macrophages (Cat. No. CL-0190) were obtained from Procell Life Science & Technology Co., Ltd. (Wuhan, China). Cell viability was assessed using the Cell Counting Kit-8 (CCK-8; Cat. No. CK04-1000T, Dojindo Laboratories, Kumamoto, Japan). Intracellular reactive oxygen species (ROS) levels were measured with a ROS detection kit (Cat. No. KGA7308-100, KeyGEN BioTECH, Nanjing, China), and nuclei were counterstained with Hoechst 33342 (Cat. No. C-1022, Beyotime Biotechnology, Shanghai, China). Fluorescence images were acquired using a high-content imaging system (Operetta CLS, PerkinElmer, Waltham, MA, USA). Nitric oxide (NO) production was determined using a Griess reagent kit (Cat. No. S0021S, Beyotime Biotechnology, Shanghai, China). The levels of TNF-α and IL-6 in culture supernatants were quantified using commercial ELISA kits (Mouse TNF-alpha Fast Pro ELISA Kit, Cat. No. RK05228; Mouse IL-6 Fast Pro ELISA Kit, Cat. No. RK05213; ABclonal, Wuhan, China). Soluble AKR1B1 protein levels in the CETSA were measured using a mouse aldose reductase (AKR1B1) ELISA kit (Cat. No. M176825-96T, Solarbio, Beijing, China). AKR1B1 enzymatic activity was determined using the Aldose Reductase Activity Kit (Colorimetric; Cat. No. ab273276, Abcam, Cambridge, UK). All commercial kits were used according to the manufacturers’ instructions.

^1^H NMR and ^13^C NMR spectra were recorded on a Bruker AVANCE 600 spectrometer (Bruker Inc., Fällanden, Switzerland) operating at 600 MHz for ^1^H and 151 MHz for ^13^C in DMSO-d_6_ solvent. High-resolution electrospray ionization mass spectrometry (HR-ESI-MS) data were acquired on a Thermo Scientific Orbitrap Eclipse Tribri Mass Spectrometer (Thermo Fisher Scientific, Waltham, MA, USA). Fourier-transform infrared (FT-IR) spectra were collected on a Bruker INVENIO FT-IR spectrometer (Bruker Inc., Fällanden, Switzerland) in ATR mode. Specific optical rotation was tested with an Autopol IV automatic polarimeter (Rudolph Research Analytical, Hackettstown, NJ, USA). Melting point was determined via differential scanning calorimetry (DSC) on a Discovery DSC 250 instrument (TA Instruments, New Castle, DE, USA). Full original raw spectra of CGAA and CGL are deposited in the Supplementary Materials; complete spectroscopic and physical data are compiled in Section 4.2.

### 4.2. Synthesis and Characterization of Chlorogenic Acid Derivatives

**Scheme 1:**
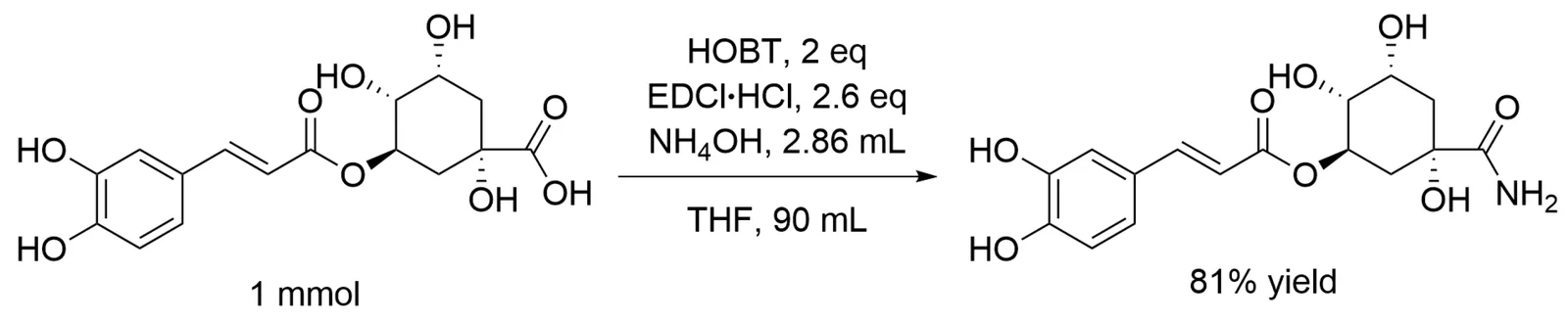
Synthetic route of CGAA from natural CGA via one-step amidation.

CGAA was synthesized from natural chlorogenic acid (CGA) through a one- -step selective amidation of the quinic acid carboxyl group (Scheme 1). CGA (1 mmol) was dissolved in anhydrous tetrahydrofuran (THF, 90 mL). N-Hydroxybenzotriazole (HOBT, 2.0 equiv.) and 1-ethyl-3-(3-dimethylaminopropyl)carbodiimide hydrochloride (EDCI·HCl, 2.6 equiv.) were sequentially added, and the reaction mixture was stirred at room temperature for 16 h to activate the carboxyl group. Aqueous ammonia (29.6%, 2.86 mL) was then added dropwise, and the reaction was allowed to proceed for an additional 1 h. The reaction was quenched by adjusting the pH to approximately 3 with 1 M HCl. The mixture was extracted with ethyl acetate, and the combined organic layers were washed sequentially with pH 7 phosphate buffer and saturated brine, dried over anhydrous Na_2_SO_4_, and concentrated under reduced pressure. The crude product was purified by silica gel column chromatography using CH_2_Cl_2_/MeOH (94:6, *v*/*v*) as eluent to afford CGAA as a pale-yellow solid in 81% isolated yield.

**Scheme 2:**
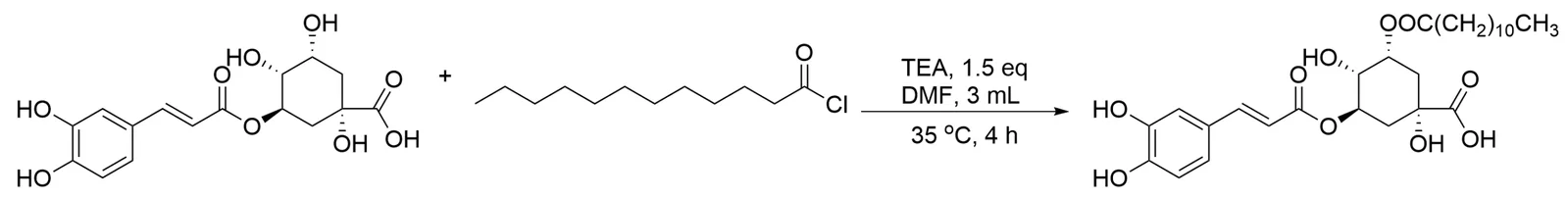
Synthetic route of CGL from natural CGA.

CGL was synthesized according to a previously reported procedure (Scheme 2) [27]. CGA (1 mmol) was dissolved in anhydrous N,N-dimethylformamide (DMF, 3 mL), followed by the addition of triethylamine (TEA, 3 mmol). After stirring at 35 °C for 4 h, lauroyl chloride (1 mmol) was added portion-wise, and the reaction mixture was stirred for an additional 4 h at room temperature. The resulting precipitate was collected by filtration and washed successively with cold 0.5 mol/L acetic acid and deionized water. The crude product was purified by sequential recrystallization from petroleum ether/acetone (10:3, *v*/*v*) at 6 °C, then from n-hexane-ethanol (2:1, *v*/*v*) at 18 °C to obtain CGL as a pale-yellow solid in 80% isolated yield.

#### Spectroscopic and Physical Data

CGAA: Pale-yellow solid, isolated yield 81%; purity 99.2% (determined by HPLC, area normalization method); m.p. 204.5 °C; ${\left[\alpha \right]}_{D}^{25}$ = −25.7 (c =1.0, MeOH). ^1^H NMR (600 MHz, DMSO-d_6_) δ 9.58 (s, 1H), 9.15 (s, 1H), 7.47 (d, *J* = 15.9 Hz, 1H), 7.21 (d, *J* = 3.0 Hz, 1H), 7.14 (d, *J* = 3.0 Hz, 1H), 7.04 (d, *J* = 2.2 Hz, 1H), 7.00 (dd, *J* = 8.2, 2.1 Hz, 1H), 6.76 (d, *J* = 8.1 Hz, 1H), 6.24 (d, *J* = 15.9 Hz, 1H), 5.60 (d, *J* = 4.3 Hz, 1H), 5.51 (s, 1H), 5.27–5.20 (m, 1H), 5.03 (d, *J* = 5.9 Hz, 1H), 4.07 (t, *J* = 3.4 Hz, 1H), 3.58–3.53 (m, 1H), 1.97–1.90 (m, 2H), 1.88–1.83 (m, 1H), 1.78 (dt, *J* = 14.4, 3.3 Hz, 1H). ^13^C NMR (151 MHz, DMSO-d_6_) δ 176.7, 166.7, 148.8, 146.0, 145.3, 126.1, 121.7, 116.2, 115.2, 112.0, 76.5, 72.8, 71.4, 70.9, 39.3, 37.8. FTIR: ν_max_ 3400–3200 (br, O-H/N-H), 2930 (C-H), 1655 (s, amide I, C=O), 1602 (aromatic C=C), 1525 (amide II, N-H), 1200–1000 (C-O) cm^−1^. HRMS (ESI): *m*/*z* calcd for C_16_H_18_NO_8_ [M + H]^+^ 354.1111, found 354.1186.

CGL: Pale-yellow solid, isolated yield 80%. ^1^H NMR (600 MHz, DMSO-d_6_) δ 12.79 (s, 1H), 9.57 (s, 1H), 9.13 (s, 1H), 7.46 (d, *J* = 15.9 Hz, 1H), 7.03 (d, *J* = 2.1 Hz, 1H), 6.98 (dd, *J* = 8.2, 2.1 Hz, 1H), 6.77 (d, *J* = 8.1 Hz, 1H), 6.18 (d, *J* = 15.9 Hz, 1H), 5.16 (td, *J* = 8.4, 4.0 Hz, 1H), 4.92 (d, *J* = 5.6 Hz, 1H), 4.54 (d, *J* = 4.5 Hz, 1H), 4.03 (t, *J* = 4.1 Hz, 1H), 3.60–3.57 (m, 1H), 2.27–2.20 (m, 5H), 1.83 (dd, *J* = 13.4, 8.8 Hz, 1H), 1.55–1.48 (m, 2H), 1.24 (s, 16H), 0.85 (t, *J* = 6.9 Hz, 3H). ^13^C NMR (151 MHz, DMSO-d6) δ172.9, 172.2, 166.4, 148.9, 146.0, 145.6, 126.0, 121.8, 116.2, 115.2, 114.6, 79.6, 71.2, 70.3, 67.7, 36.2, 34.3, 34.0, 31.8, 29.5, 29.4, 29.2, 29.2, 28.8, 24.7, 22.6, 14.4.

### 4.3. Cell Culture and Treatment

RAW264.7 murine macrophages were cultured in high-glucose Dulbecco’s modified Eagle’s medium (DMEM) supplemented with 10% (*v*/*v*) fetal bovine serum (FBS), 1% L-glutamine, and 1% penicillin-streptomycin solution. Cells were maintained at 37 °C in a humidified incubator with 5% CO_2_. For inflammation induction, cells were pretreated with CGAA, CGA, or CGL at concentrations of 10, 25, 50, or 100 μM for 2 h, followed by stimulation with lipopolysaccharide (LPS, 1 μg/mL) for an additional 24 h. Epalrestat (50 μM), a clinically approved selective AKR1B1 inhibitor, was set as the positive control to compare the anti-inflammatory efficacy of CGAA with a marketed AKR1B1 inhibitor. The concentrations used in subsequent functional assays (10, 25, 50, and 100 μM) were selected based on the non-cytotoxic concentration window determined by the CCK-8 assay.

### 4.4. Cell Viability Assay

Cell viability was determined using the Cell Counting Kit-8 (CCK-8) assay. RAW264.7 macrophages were seeded into 96-well plates at a density of 1 × 10^5^ cells/well in 100 μL of complete DMEM and allowed to attach overnight. Cells were initially exposed to a broad concentration range of 10–1280 μM to evaluate the intrinsic cytotoxicity of the three compounds. Based on the observation that CGL exhibited marked cytotoxicity above 160 μM, a refined concentration series of 20, 40, 60, 80, and 100 μM was selected for quantitative comparison of cell viability and determination of the non-cytotoxic concentration window used in subsequent cellular experiments. After treatment, 10 μL of CCK-8 reagent was added to each well, and the plates were incubated at 37 °C for 2 h. Absorbance was measured at 450 nm using a microplate reader, and cell viability was expressed as a percentage relative to the untreated control group.

### 4.5. Measurement of NO, ROS, and Pro-Inflammatory Cytokines

Raw264.7 macrophages were seeded in appropriate culture plates and treated as described in Section 4.3. After pretreatment with CGAA, CGA, or CGL for 2 h, cells were stimulated with LPS (1 μg/mL) for 24 h. Nitric oxide (NO) content in cell culture supernatants was determined using the Griess reagent system according to the manufacturer’s protocol. Intracellular reactive oxygen species (ROS) levels were assessed using the fluorescent probe 2′,7′-dichlorodihydrofluorescein diacetate (DCFH-DA). For cytokine quantification, the concentrations of tumor necrosis factor-α (TNF-α) and interleukin-6 (IL-6) in supernatants were measured using commercial enzyme-linked immunosorbent assay (ELISA) kits, following the manufacturer’s instructions. All measurements were performed in triplicate.

### 4.6. Cellular Thermal Shift Assay (CETSA)

A temperature-gradient CETSA was performed to assess ligand-induced thermal stabilization of AKR1B1 [23]. RAW264.7 cells were seeded in 24-well plates at a density of 1 × 10^5^ cells/mL and allowed to adhere for 24 h. Cells were then treated with CGAA, CGA, or CGL (50 μM), or vehicle control for 24 h. After treatment, cells were harvested and aliquots were heated at 4, 45, 60, 75, or 90 °C for 3 min, followed by rapid cooling on ice. Cells were subsequently lysed, and total protein was extracted. Protein concentration was determined using the BCA method. Soluble AKR1B1 levels were quantified using a commercial ELISA kit according to the manufacturer’s instructions. Relative AKR1B1 protein content at each temperature was normalized to the corresponding 4 °C sample of the same treatment group.

### 4.7. Drug Affinity Responsive Target Stability (DARTS) Assay

The DARTS assay was performed to evaluate the direct interaction between the test compounds and recombinant human AKR1B1 protein based on ligand-induced resistance to proteolytic digestion [23]. Recombinant AKR1B1 protein (10 μg/mL) was incubated with CGAA, CGA, or CGL at final concentrations of 10, 25, 50 and 100 μM at 37 °C for 20 min. The protein–compound mixtures were then subjected to digestion with 0.1% trypsin for 10 min at room temperature, and the reaction was terminated by the addition of 10% fetal bovine serum. The residual AKR1B1 enzymatic activity after proteolysis was determined using a commercial AKR1B1 activity assay kit by monitoring NADPH consumption at 340 nm with a microplate reader. Parallel compound-treated samples without trypsin digestion were included to exclude direct inhibition of AKR1B1 activity by the compounds during the DARTS assay. The residual activity of the trypsin-digested samples was expressed relative to the corresponding non-digested controls. Increased residual enzymatic activity after trypsin treatment was interpreted as enhanced proteolytic resistance resulting from compound binding to AKR1B1. All measurements were performed in triplicate. An activity-based readout was selected in the DARTS assay to quantify functionally intact AKR1B1 remaining after proteolysis, whereas CETSA measured the abundance of soluble AKR1B1 protein after thermal denaturation.

### 4.8. Aldose Reductase (AKR1B1) Enzyme Activity Assay

This enzyme activity assay was performed to quantitatively compare the direct inhibitory potency of CGAA, CGA and CGL against AKR1B1 at the protein level, and further calculate the IC50 values to evaluate their target inhibitory potency. The inhibitory effect of test compounds on AKR1B1 enzyme activity was determined using a commercial colorimetric assay kit. Briefly, recombinant AKR1B1 protein was incubated with gradient concentrations of CGAA, CGA, or CGL (0.1–16 μM) at 37 °C. The absorbance at 340 nm was continuously monitored for 60 min to track NADPH consumption, as NADPH exhibits characteristic absorbance at 340 nm and its reduction directly reflects the catalytic activity of AKR1B1. Relative enzyme activity was calculated based on the rate of absorbance decrease. The half-maximal inhibitory concentration (IC_50_) values were fitted and determined using GraphPad Prism 9 software.

### 4.9. Molecular Docking

Molecular docking simulation was adopted to reveal the atomic-level structural basis for the different binding affinity of CGA, CGL and CGAA with AKR1B1, and to elucidate how carboxyl-amidation enhances ligand–protein intermolecular interactions between compound and protein pocket. The molecular docking simulations were performed using Schrödinger Maestro software (version 2018-1, Schrödinger, LLC, New York, NY, USA). The three-dimensional structures of CGA, CGL, and CGAA were prepared using the LigPrep module, including energy minimization, charge assignment, addition of polar and non-polar hydrogens, generation of possible ionization states, and definition of rotatable bonds. Structural optimization of the ligands was carried out using the OPLS3 force field. The crystal structure of human AKR1B1 (PDB ID: 8FH5) was retrieved from the RCSB Protein Data Bank (www.rcsb.org). The protein was prepared using the Protein Preparation Wizard module in Schrödinger Maestro, which included hydrogenation, removal of solvent molecules, and assignment of bond orders. Molecular docking was then performed using the Glide module with the generated receptor grid file. The binding poses and non-covalent interactions (hydrogen bonds, hydrophobic interactions, and π-stacking) were visualized and analyzed using PyMOL (version 3.1.8, Schrödinger, LLC, New York, NY, USA).

### 4.10. Statistical Analysis

All experiments were performed in biological triplicate, and all quantitative data are presented as mean ± standard deviation (SD). Statistical analyses were carried out using GraphPad Prism 9 software (GraphPad Software, San Diego, CA, USA). One-way analysis of variance (ANOVA) coupled with Tukey’s multiple comparison test was applied to assess intergroup differences. A threshold of *p* < 0.05 was defined as statistically significant.

## 5. Conclusions

This work successfully synthesized CGAA via selective quinic acid carboxyl amidation of natural CGA, retaining the bioactive polyphenol backbone without chiral racemization. Cellular assays confirmed CGAA possesses optimal cellular safety while exerting stronger antioxidant and anti-inflammatory effects than both the parent CGA and the ester derivative CGL. Temperature-gradient CETSA, DARTS, enzymatic assays, and molecular docking collectively demonstrated that the newly introduced amide group forms an additional hydrogen bond with ILE-260 within the AKR1B1 catalytic pocket, thereby elevating ligand binding affinity and inhibition potency (IC_50_ = 7.85 μM). Distinct from previously reported CGA ester and phenolic-amide derivatives, CGAA achieves dual advantages of low cytotoxicity and potent AKR1B1 suppression. Although the current research is limited to in vitro macrophage models and lacks in vivo validation as well as comprehensive macrophage polarization profiling, this carboxyl-amidation strategy provides a rational approach for developing low-toxic polyphenolic AKR1B1 antagonists for the intervention of inflammatory disease.

## Data Availability

The original contributions presented in this study are included in the article/Supplementary Materials. Further inquiries can be directed to the corresponding author.

## Supplementary Materials

The following supporting information can be downloaded at https://www.mdpi.com/article/10.3390/molecules31183247/s1: Figure S1: ^1^H-NMR spectrum of CGAA; Figure S2: ^13^C-NMR spectrum of CGAA; Figure S3: HSQC spectrum of CGAA; Figure S4: HMBC spectrum of CGAA; Figure S5: FT-IR spectrum of CGAA; Figure S6: HRMS(ESI-MS) spectrum of CGAA; Figure S7: HPLC chromatogram of CGAA; Figure S8: 1H-NMR spectrum of CGL; Figure S9: 13C-NMR spectrum of CGL.

## Abbreviations

The following abbreviations are used in this manuscript:

AKR1B1	Aldo-keto reductase family 1 member B1
CCK-8	Cell Counting Kit-8
CETSA	Cellular thermal shift assay
CGA	Chlorogenic acid
CGAA	Chlorogenic acid amide derivative
CGL	Chlorogenic acid laurate
DARTS	Drug affinity responsive target stability
DCFH-DA	2′,7′-Dichlorodihydrofluorescein diacetate
ELISA	Enzyme-linked immunosorbent assay
FTIR	Fourier-transform infrared spectroscopy
HMBC	Heteronuclear multiple bond correlation
HRMS	High-resolution mass spectrometry
HSQC	Heteronuclear single quantum correlation
IL-6	Interleukin-6
LPS	Lipopolysaccharide
NO	Nitric oxide
ROS	Reactive oxygen species
TNF-α	Tumor necrosis factor-α
