# Supplementary material for: Carboxyl Amidation of Chlorogenic Acid Improves Anti-Inflammatory Activity and Biosafety via Potent AKR1B1 Inhibition in LPS-Induced Macrophages
Source: Molecules. 2026 Sep 14;31(18):3247. doi: 10.3390/molecules31183247 (PMC13609249; doi:10.3390/molecules31183247)
Supplement: Supplementary file 1 [file molecules-31-03247-s001.zip › molecules-4530403-supplementary.pdf]

Novel Chlorogenic Acid Amide (CGAA) Exerts Potent Anti-Inflammatory and Antioxidant Effects by Targeting AKR1B1 in LPS-Stimulated RAW264.7 Macrophages

Faliu Yang, Chunmei Guang, Huan Chen and Hongwei Hou

Table of Contents

Figure S1. ^{1}H-NMR spectrum of CGAA S2
Figure S2. ^{13}C-NMR spectrum of CGAA S2
Figure S3. HSQC spectrum of CGAA S3
Figure S4. HMBC spectrum of CGAA S3
Figure S5. FT-IR spectrum of CGAA S4
Figure S6. HRMS (ESI-MS) spectrum of CGAA S4
Figure S7. HPLC chromatogram of CGAA S4
Figure S8. ^{1}H-NMR spectrum of CGL S5
Figure S9. ^{13}C-NMR spectrum of CGL S5

^{1}H NMR

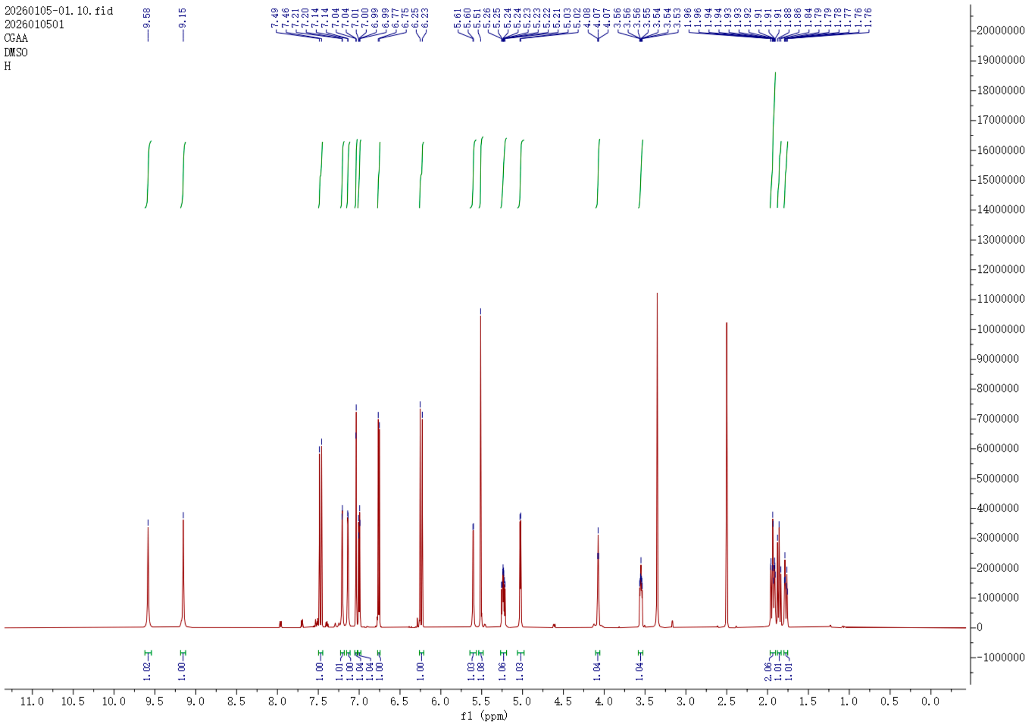

Figure S1. ^{1}H-NMR spectrum of CGAA

^{13}C NMR

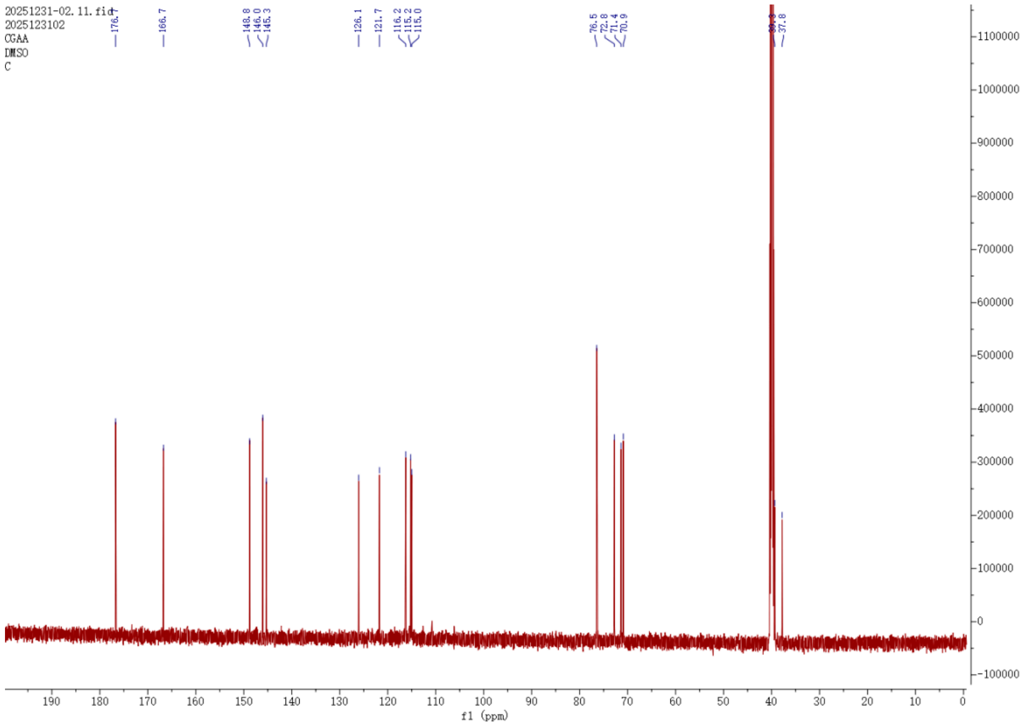

Figure S2. ^{13}C-NMR spectrum of CGAA

HSQC

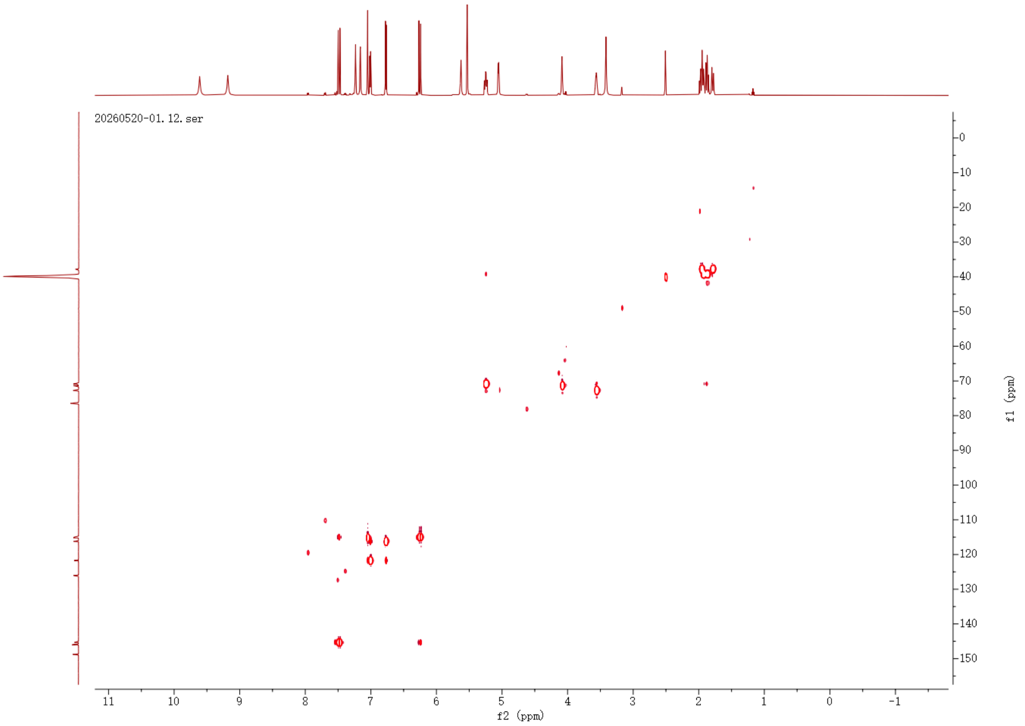

Figure S3. HSQC spectrum of CGAA

HMBC

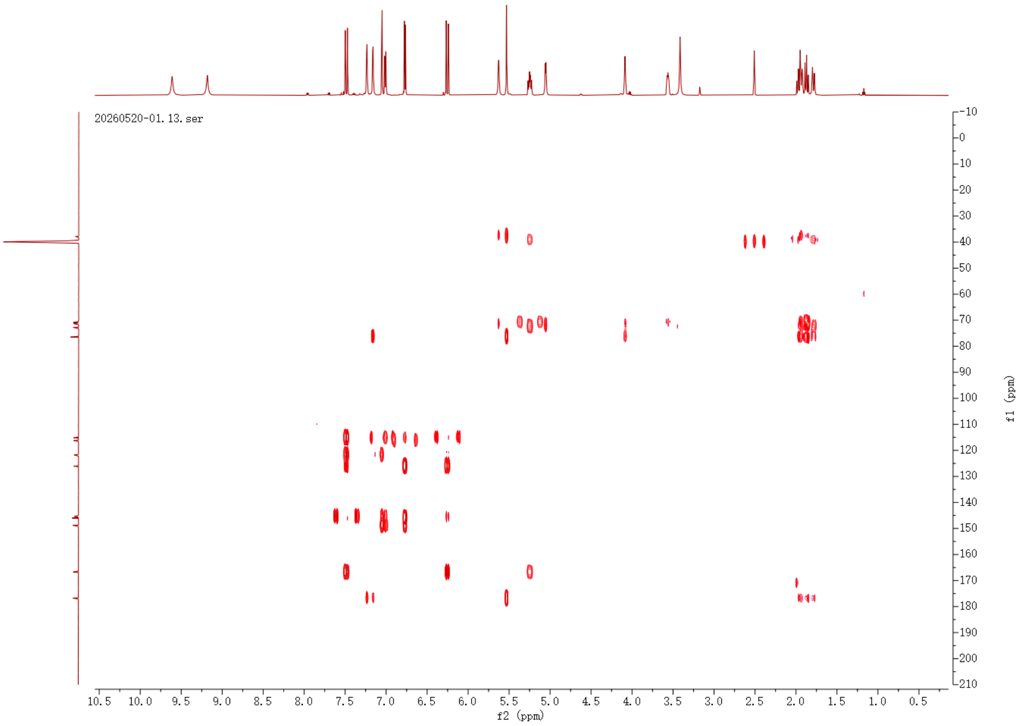

Figure S4. HMBC spectrum of CGAA

FTIR

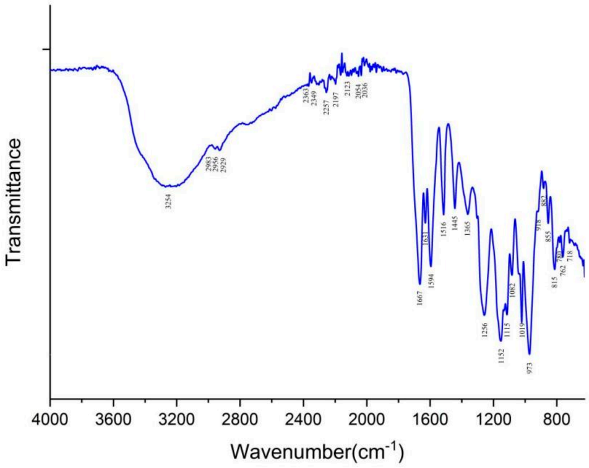

Figure S5. FT-IR spectrum of CGAA

HRMS

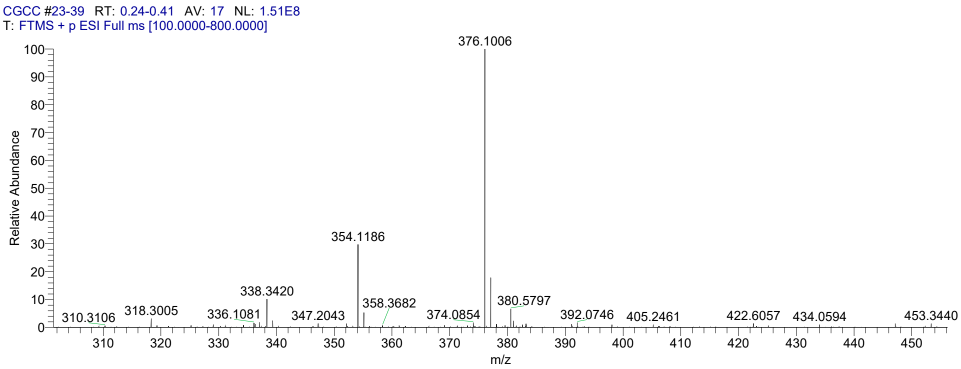

Figure S6. HRMS (ESI-MS) spectrum of CGAA

HPLC chromatogram of CGAA

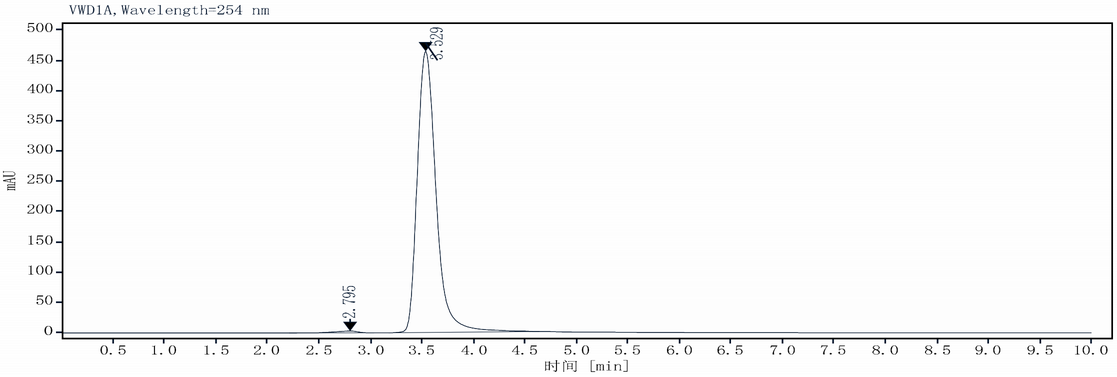

Figure S7. HPLC chromatogram of CGAA showing a purity of 99.2% (area normalization method)

^{1}H NMR

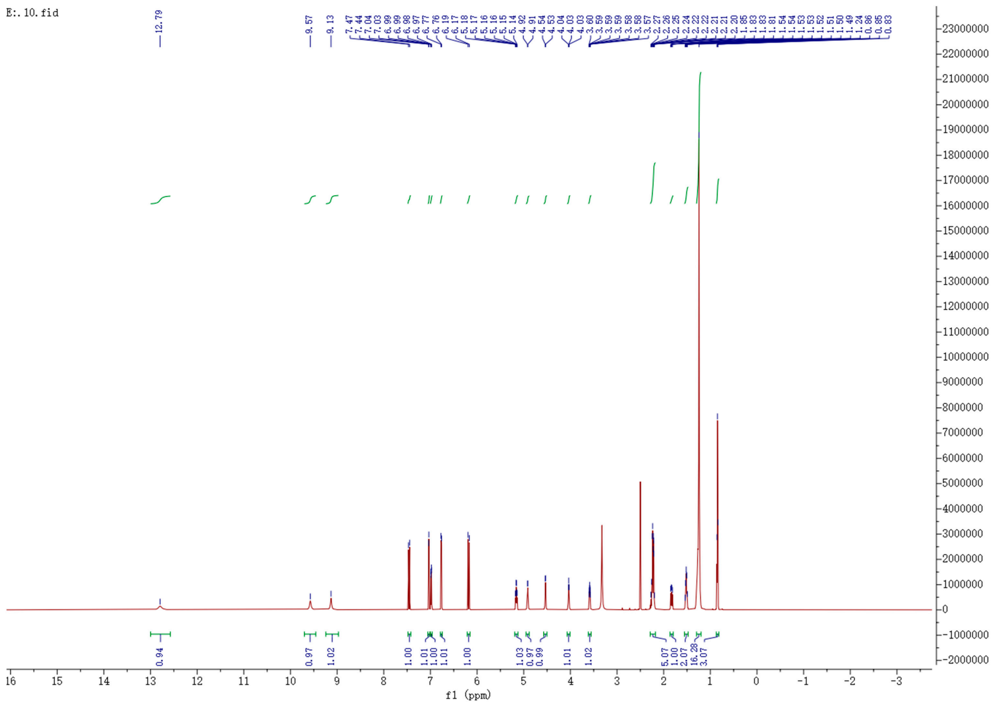

Figure S8. ^{1}H-NMR spectrum of CGL

^{13}C NMR

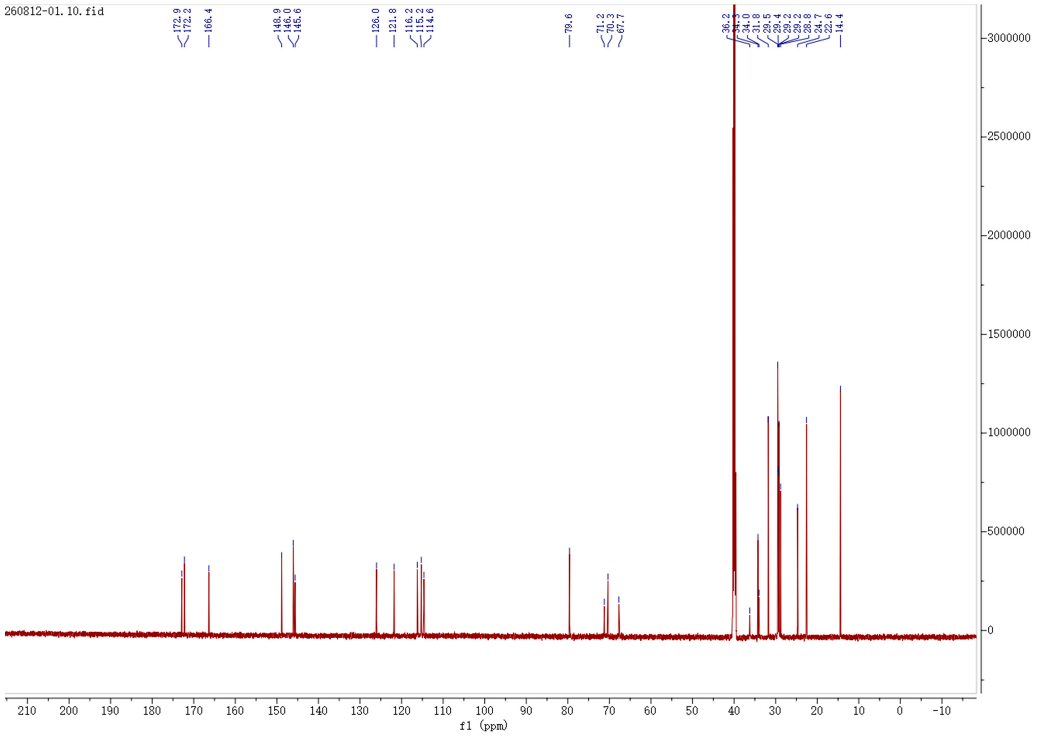

Figure S9. ^{13}C-NMR spectrum of CGL
